# Approximate Single-Diode Photovoltaic Model for Efficient *I*-*V* Characteristics Estimation

**DOI:** 10.1155/2013/230471

**Published:** 2013-11-05

**Authors:** Jieming Ma, Ka Lok Man, T. O. Ting, Nan Zhang, Sheng-Uei Guan, Prudence W. H. Wong

**Affiliations:** ^1^Department of Computer Science, University of Liverpool, Ashton Building, Ashton Street, Liverpool L69 3BX, UK; ^2^Xi'an Jiaotong-Liverpool University, 111 Ren'ai Road, Jiangsu, Suzhou 215123, China

## Abstract

Precise photovoltaic (PV) behavior models are normally described by nonlinear analytical equations. To solve such equations, it is necessary to use iterative procedures. Aiming to make the computation easier, this paper proposes an approximate single-diode PV model that enables high-speed predictions for the electrical characteristics of commercial PV modules. Based on the experimental data, statistical analysis is conducted to validate the approximate model. Simulation results show that the calculated current-voltage (*I*-*V*) characteristics fit the measured data with high accuracy. Furthermore, compared with the existing modeling methods, the proposed model reduces the simulation time by approximately 30% in this work.

## 1. Introduction

Photovoltaic (PV) power market has grown rapidly in the last decade owing to the deterioration of the environmental quality and the escalation of fossil fuel price. Before installing a PV system, a good performance estimation of the adopted PV generators is necessary since the initial cost of the system is pretty high [1, 2]. Unfortunately, although PV generators always work under the operating environment far from the standard test conditions (STCs), PV manufacturers usually only list limited technical data measured at STCs, such as maximum power (*P*_max⁡_), voltage at *P*_max⁡_ (*V*_mp_), current at *P*_max⁡_ (*I*_mp_), short circuit current (*I*_sc_), and open-circuit voltage (*V*_oc_). For this reason, a reliable and flexible PV model that enables an accurate estimation of the PV generated electricity towards various operating conditions is of significance in the design phase.

Among numerous modeling approaches in the literature, the most widely used circuit-based PV model is the single-diode model (SDM), which consists of a series resistance (*R*_*s*_), a shunt resistance (*R*_*p*_), and a linear independent current source in parallel to a diode. The more accurate double-diode model (DDM) is available in [3]. It takes into consideration the recombination loss at the space depletion region of solar cells. In [4], the electrical characteristics of the multicrystalline solar cells are analyzed by a three-diode model (TDM), which further takes into account the influence of grain boundaries and leakage current through the peripheries. Although the DDM and TDM have certain advantages, the extra diodes increase the computational complexity. Accordingly, the SDM is considered to feature a good compromise between simplicity and accuracy. This may be the most likely reason why commercial simulation tools (e.g., PSIM [5] and PVsyst [6]) frequently apply the SDM in combination with the intricate dependence of the electric current on weather-related and environment factors, such as the ambient temperature (*T*) and the irradiance (*G*).

More recently, there is an increasing need for high-speed performance estimation as PV models are frequently used to aid real-time optimization of PV energy [7–16]. Ignoring the effect of the resistance is a typical approach to reduce the complexity of PV models. In [17], Mahmoud proposes the simplified single-diode model (SSDM) which removes the *R*_*p*_ from the general SDM. The further simplified single-diode model (FSSDM), also known as the ideal single-diode model (ISDM), neglects the *R*_*s*_ and *R*_*p*_ as well. Albeit their simplicity, accurate estimation of the electrical characteristics is not guaranteed [14]. Furthermore, tedious iterative root finding methods (e.g., Newton-Raphson method) are still needed in the SDM and SSDM to solve the implicit transcendental equations. In [18, 19], Jain et al. proposed Lambert-W function-based SDM which enables the solutions to be exact, explicit, and straightforward and is not necessary to ignore resistance effects. However, that model does not intrinsically reduce the complexity because the root of the Lambert W-function can only be calculated by using iterative approximations [20].

This paper proposes a simple yet accurate approximate single-diode model (ASDM) aiming to overcome the limitations in the existing simplified SDMs. The exponential diode behavior is approximated via continuous least squares approximation (CLSA), which permits designers or engineers to predict the current *I* by solving a closed-form expression. Only a simple numerical root-finding algorithm is required to determine the parameters of the ASDM. The accuracy and computational speed improvements are demonstrated by the simulation and experimental results.

The rest of the paper is organized as follows. In Section 2, we give a brief introduction to single-diode PV models. In Section 3, the proposed approximate model as well as its corresponding parameter estimation methods is presented. The accuracy and efficiency of the proposed method are validated in Section 4. This is followed by conclusions in Section 5.

## 2. Modeling of PV Modules

**Figure 1:**
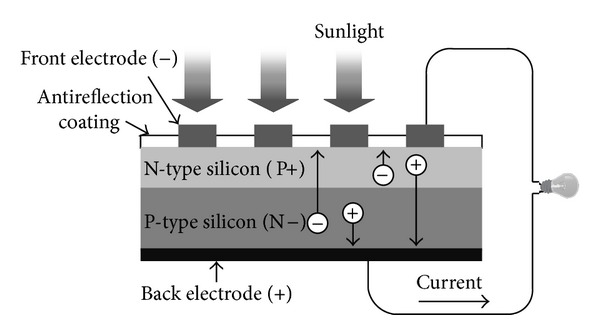
The basic structure of a silicon-based PV cell and its working mechanism.

PV cells are made of a variety of semiconductor materials using different manufacturing processes. The working principle of PV cells is essentially based on the PV effect, which refers to the generation of a potential difference at the P-N junction in response to visible or other radiation.  Figure 1 roughly demonstrates the basic structure of a silicon-based PV cell and its working mechanism. The PV cell consists of a thin layer of bulk Si or a thin Si film connected to electric terminals [21]. A thin metallic grid is connected on the top surface of the semiconductor. On the other side, the thin semiconductor layer is specially treated to form the P-N junction.

PV module is a particular case of a series connected PV cells. When a PV module is exposed to light, the semiconductor materials absorb photons and accordingly charge carriers are generated. These carriers are separated by the P-N junction electric field and an electric current *I*_pv_ then flows through the external circuit. By eliminating the PV effect, a PV module behaves like a conventional diode that does not depend on any light parameters. The Shockley diode equation is generally used to describe the current flowing through the diode *I*_*d*_:

(1) $$\begin{array}{c} I_{d}=I_{o}\left(e^{V_{D}/nN_{s}V_{t}}-1\right). \end{array}$$

In (1), *V*_*D*_ represents the electrical potential difference between the two ends of the diode. *I*_*o*_ is the reverse saturation current, and *n* is the diode ideality factor. *V*_*t*_ denotes the thermal voltage of the PV module with *N*_*s*_ cells connected in series, and its value can be estimated as a function of *T*:

(2) $$\begin{array}{c} V_{t}=\frac{kT}{q}, \end{array}$$

where *k* and *q* represent the Boltzmann constant (1.380650 × 10^−23^ J/K) and the electron charge (1.602176 × 10^−19^ C), respectively.

A simple approach, describing the value of *I*, is to assume that the superposition principle holds; that is, the total characteristic is the sum of the dark and illuminated characteristics [17, 21, 22]. Alternatively, the terminal current *I* is equal to the *I*_pv_ minus the current diverting through the diode:

(3) $$\begin{array}{c} I=I_{\text{pv}}-I_{o}\left(e^{V/nN_{s}V_{t}}-1\right). \end{array}$$

**Figure 2:**
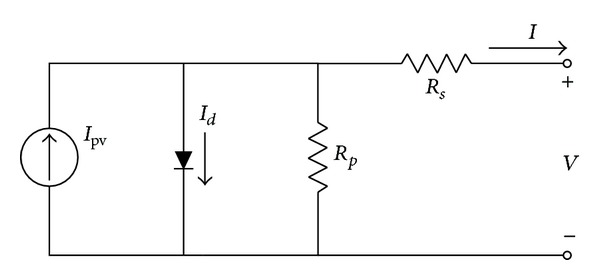
Circuit diagram of the SDM.

The modeling methods described so far consider the ideal behavior of PV modules based on a current source in parallel with an ideal diode. The SDM, whose circuit diagram is shown in Figure 2, improves the ideal model by recognizing *R*_*s*_ and *R*_*p*_. Equation (4) mathematically describes the *I-V* characteristics of the SDM:

(4) $$\begin{array}{c} I=I_{\text{pv}}-I_{o}\left(e^{\left(V+IR_{s}\right)/nN_{s}V_{t}}-1\right)-\frac{V+IR_{s}}{R_{p}}. \end{array}$$

## 3. PV Model Approximation

### 3.1. Function Approximation

Function approximation provides an approach to represent a complicated function *f*(*x*) (*f*(*x*) ∈ *C*[*a*, *b*]) by an easier form *ϕ*(*x*; *a*_0_, *a*_1_,…, *a*_*n*_), where *a*_0_, *a*_1_,…, *a*_*n*_ are parameters to be determined so as to achieve the best approximation of *f*(*x*). The term least squares describes a frequently used means to solving overdetermined or inexactly specified equations (e.g., transcendental functions, integrals, and solutions of differential or algebraic equations) in an approximate sense [23]. Normally, least squares approximation (LSA) can be viewed as finding proper coefficients *a*_0_, *a*_1_,…, *a*_*n*_ so as to

(5) $$\begin{array}{c} \text{minimize}{||f\left(x\right)-\phi \left(x;a_{0},a_{1},\ldots ,a_{n}\right)||}_{2}, \end{array}$$

where *ϕ*(*x*; *a*_0_, *a*_1_,…, *a*_*n*_) is usually a polynomial *P*_*n*_(*x*) of degree at most *n*:

(6) $$\begin{array}{c} P_{n}=a_{0}+a_{1}x+\cdot \cdot \cdot +a_{n}x^{n}=\sum_{k=0}^{n}a_{k}x^{k}. \end{array}$$

The approximation problem might be regarded as a process of minimizing the error *E*, which is given in (7):

(7) $$\begin{array}{c} E\equiv E\left(a_{0},a_{1},\ldots ,a_{n}\right)=\int_{b}^{a}{\left(f\left(x\right)-P_{n}\left(x\right)\right)}^{2}dx. \end{array}$$

By applying the derivative to (7), we get

(8) $$\begin{array}{c} \frac{\partial E}{\partial a_{j}}=-2\int_{a}^{b}x^{j}f\left(x\right)dx+2\sum_{k=0}^{n}a_{k}\int_{a}^{b}x^{j+k}dx. \end{array}$$

With the aim of finding real coefficients *a*_0_, *a*_1_,…, *a*_*n*_, a necessary condition that should be considered is

(9) $$\begin{array}{c} \frac{\partial E}{\partial a_{j}}=0,\;j=0,1,\ldots ,n. \end{array}$$

After substituting (9) into (7), the linear normal equations, expressed by (10), can be derived to solve the unknown coefficients *a*_0_, *a*_1_,…, *a*_*n*_. It has been proven that the normal equations always have a unique solution provided *f*(*x*) ∈ *C*[*a*, *b*] [24].

(10) $$\begin{array}{c} \int_{a}^{b}x^{j}f\left(x\right)dx=\sum_{k=0}^{n}a_{k}\int_{a}^{b}x^{j+k}dx,\;\text{for}\;\text{each}\;\;j=0,1,\ldots ,n. \end{array}$$

All the above approximation process is called continuous least square approximation (CLSA) in the field of applied mathematics.

### 3.2. Approximate Single-Diode Model (ASDM)

In a typical SDM, the analytical expression of the forward *I-V* characteristics contains a transcendental function for predicting the value of *I*_*d*_, which is formulated as

(11) $$\begin{array}{c} I_{d}=I_{o}\left(e^{\left(V+IR_{s}\right)/nN_{s}V_{t}}-1\right). \end{array}$$

Assuming that the parameters are constant at a certain test condition, the value of *I* varies directly with the reference *V*. Let *m* = *R*_*s*_/*nN*_*s*_ *V*_*t*_; then *I*_*d*_ can be rewritten as a function of *I*:

(12) $$\begin{array}{c} I_{d}\left(I\right)=I_{o}e^{mV/R_{s}}\cdot e^{mI}-I_{o}. \end{array}$$

CLSA provides a paradigm that simplifies the transcendental part of (12) into a polynomial of degree 1:

(13) $$\begin{array}{c} e^{mI}≅a_{0}+a_{1}I. \end{array}$$

By using the linear normal equations, namely, (10), the values of *a*_0_ and *a*_1_ can be solved. The detailed deduction process is given in the appendix, and their exact mathematical expressions are given in (14):

(14) $$\begin{array}{c} a_{0}=-\frac{2}{mI_{\max}^{2}}\left[\left(I_{\max}-\frac{3}{m}\right)e^{mI_{\max}}+\left(2I_{\max}+\frac{3}{m}\right)\right],\\ a_{1}=\frac{12}{mI_{\max}^{3}}\left[\left(\frac{I_{\max}}{2}-\frac{1}{m}\right)e^{mI_{\max}}+\left(\frac{I_{\max}}{2}+\frac{1}{m}\right)\right]. \end{array}$$

Accordingly, the ASDM can be formulated as a rational function:

(15) $$\begin{array}{c} I≅\frac{I_{\text{pv}}-\left(I_{o}e^{V/nN_{s}V_{t}}\right)\cdot a_{0}-V/R_{p}}{1+\left(I_{o}e^{V/nN_{s}V_{t}}\right)\cdot a_{1}+R_{s}/R_{p}}. \end{array}$$

The methods of determining the parameters *I*_pv_, *I*_*o*_, *n*, *R*_*s*_, and *R*_*p*_ are presented in the next subsection.

### 3.3. Parameter Identification for the Proposed Model

#### 3.3.1. Analytical Methods for Predicting *I*_pv_ and *I*_*o*_

As a result of the PV effect, the photocurrent *I*_pv_ flows in a direction opposite to the forward dark current. Even when the PV module operates at short circuit, this current continues to flow and is measured as the short-circuit current *I*_sc_. From (3), it can be seen that the value of *I*_pv_ is approximately equal to the *I*_sc_ in a high-quality PV module, and thus the assumption *I*_sc_≅*I*_pv_ is often used in PV modeling. Although the spectral short current density can be determined by analytical equations in [22], the required parameters are usually not given in the manufacturer's tabular data. In view of the fact that the *I*_sc_ depends linearly on the *G* and is also slightly influenced by the *T*, the *I*_pv_ can be given by (16) [21, 25, 26]:

(16) $$\begin{array}{c} I_{\text{pv}}≅I_{\text{sc}}=\left(I_{\text{scn}}+K_{i}\Delta T\right)\frac{G}{G_{n}}, \end{array}$$

where *I*_scn_ and *G*_*n*_ are the short current and irradiance at STCs, respectively. *K*_*i*_, named short-circuit current coefficient, is a constant available in the datasheet. The difference between *T* and the standard test temperature *T*_*n*_ is denoted by Δ*T*.

The saturation current *I*_*o*_ is the small current that flows when the P-N junction is reverse biased. The dependence of *I*_*o*_ on the temperature was studied by Villalva et al. [21], in which the authors introduced (17) to predict the value of *I*_*o*_. In the expression, *K*_*v*_ is the open-circuit voltage coefficient and *V*_ocn_ represents the open circuit voltage measured at the STCs:

(17) $$\begin{array}{c} I_{o}=\frac{\left(I_{\text{scn}}+K_{i}\Delta T\right)}{e^{\left(V_{\text{ocn}}+K_{v}\Delta T\right)/nN_{s}V_{t}}-1}. \end{array}$$

#### 3.3.2. Numerical Methods for Extracting *n*, *R*_*s*_, and *R*_*p*_

The ideality factor *n* is an important parameter used to describe whether the P-N junction behaves close to or apart from the ideal case. As reported by [27], *n* and *R*_*s*_ significantly affect the shape of *I-V* curves around the maximum power point (MPP), whereas the *R*_*p*_ determines the slope of the *I-V* curve near the point *I*_sc_. With the aim of delivering a simplified calculation approach, the parameters of the ASDM are assumed to be constant and the variables *x* = (*R*_*s*_, *n*) are solved by the equation system *f*(*x*) formed by the following.

(i) The terminal current at the MPP:
  (18) $$\begin{array}{c} I_{\text{mp}}≅\frac{I_{\text{pv}}-\left(I_{o}e^{V_{\text{mp}}/nN_{s}V_{t}}\right)\cdot a_{0}-V_{\text{mp}}/R_{p}}{1+\left(I_{o}e^{V_{\text{mp}}/nN_{s}V_{t}}\right)\cdot a_{1}+R_{s}/R_{p}}. \end{array}$$
(ii) The derivative of the terminal current with respect to the voltage at the MPP:
  (19) ∂I∂V|V=Vmp,I=Imp=−(a0+a1Imp)IoeVmp/nNsVt/nNsVt+1/Rp1+a1IoeVmp/nNsVt+Rs/Rp=−ImpVmp.

In the above equation system, *a*_0_, *a*_1_, *I*_pv_, and *I*_*o*_ are represented by (14), (16), and (17) with an STC environment set. By substituting the known operating points (0, *V*_oc_) and (*I*_sc_, 0) into (15), 1/*R*_*p*_ and *R*_*s*_/*R*_*p*_ are expressed as

(20) $$\begin{array}{c} R_{p}=\frac{V_{\text{oc}}}{I_{\text{pv}}-a_{0}I_{o}e^{V_{\text{oc}}/nN_{s}V_{t}}+I_{o}}, \end{array}$$

(21) $$\begin{array}{c} \frac{R_{s}}{R_{p}}=\left(1-a_{0}-a_{1}I_{\text{sc}}\right)\frac{I_{o}}{I_{\text{sc}}}. \end{array}$$

Finally, the Newton method illustrated in [28] is capable of solving the unknowns *n* and *R*_*s*_. In the numerical computing process, the *k*th generation of variable vector *x* gets the updated vector estimate:

(22) $$\begin{array}{c} x_{k+1}=x_{k}-J_{k}^{-1}f\left(x_{k}\right), \end{array}$$

where *J*_*k*_ is the Jacobian matrix of *f*(*x*_*k*_). Other parameters as well as *I* can be recovered by using (14)–(20).

## 4. Results and Discussion

The ASDM described in this paper is compared with the physical PV models in the commercial simulation tools, such as PSIM and PVsyst. The Villalva's model [21], a famous comprehensive approach to modeling and simulation of PV arrays in the literature, is also used for comparison. These models are programmed in MATLAB, and their capability of predicting the electrical characteristics of PV modules is validated by the experimental *I-V* data extracted from the manufacturer's datasheet. Four different PV modules produced with three various manufacturing techniques, namely, MSX60 (multicrystalline), KC200 GT (multi-crystalline), SQ150-PC (mono-crystalline), and HIT Power 180 (HIT) PV modules, are utilized for verification.

Aiming to evaluate the capability of the modeling methods to fit the characteristics of PV panels, statistical analysis is performed. In this paper, the fitness of PV models is described by the root mean square error (RMSE) and the mean absolute error (MAE) as well as the relative error (RE). They are mathematically expressed by the following equations:

(23) $$\begin{array}{c} \text{RMSE}=\sqrt{\frac{1}{n}\sum_{i=1}^{n}{\left(I_{i}-\hat{I_{i}}\right)}^{2}},\\ \text{MAE}=\frac{1}{n}\sum_{i=1}^{n}\left|I_{i}-\hat{I_{i}}\right|,\\ \text{RE}=\left|1-\frac{I_{i}}{\hat{I_{i}}}\right|\times 100\%, \end{array}$$

where *I*_*i*_ and $\hat{I_{i}}$ present the simulated and measured current at the *i*th operating point among *n* measured *I-V* pairs, respectively.  Table 1 lists the parameters of PV panels by using the methods described in Section 3.3, which deliver a convenient parameter estimation method that only requires the tabular information available in the datasheet. The obtained results are extracted under a set of STCs and are assumed to be constant in other operating conditions. The obtained RMSEs for the modules working under the STCs show a good agreement between the simulation results and experimental data.

**Figure 3:**
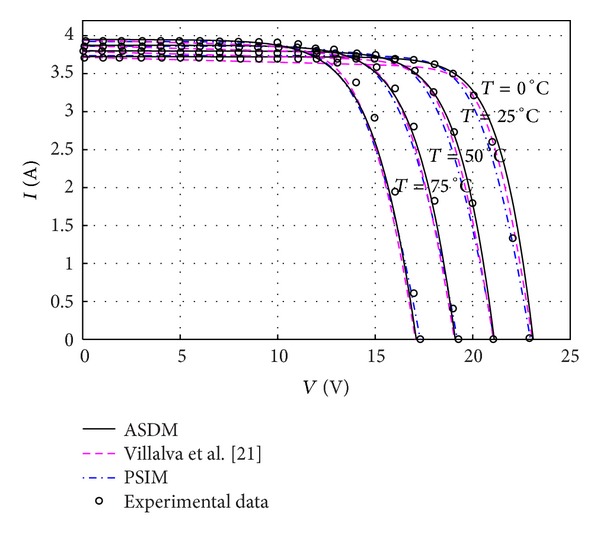
Current-voltage curves of a MSX60 PV module at various cell temperatures.

**Figure 4:**
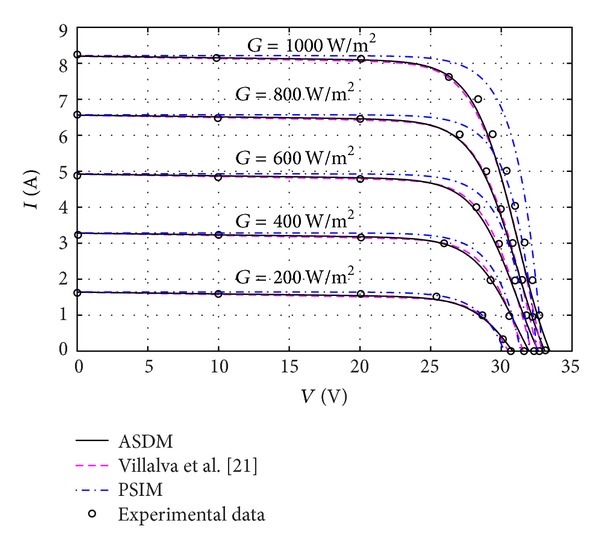
Current-voltage curves of a KC200GT PV module at various irradiance levels.

**Figure 5:**
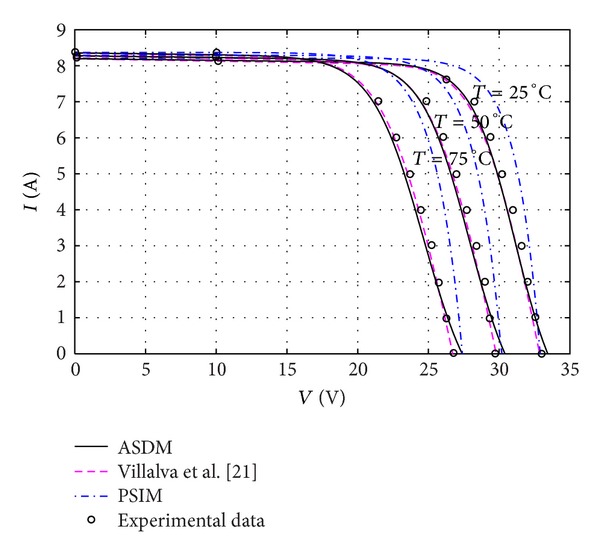
Current-voltage curves of a KC200GT PV module at various cell temperatures.

As soon as the model parameters are determined, the ASDM is able to predict the electrical characteristics of PV modules under varied atmospheric conditions. Figures 3, 4, and 5 show the *I-V* characteristics of MSX60 and KC200GT modules varying with different levels of irradiance and temperature. The simulation results of the PSIM and Villalva's models are also plotted for reference. It is interesting to see that the ASDM obtains more accurate (*I*, *V*) above the 25°C, whereas the operating points of the Villalva's model are closer to the measured data below the 25°C. Since the *I-V* curves of MSX60 at different irradiance levels are not issued in the datasheet, the related tests are not conducted in this work.

**Figure 6:**
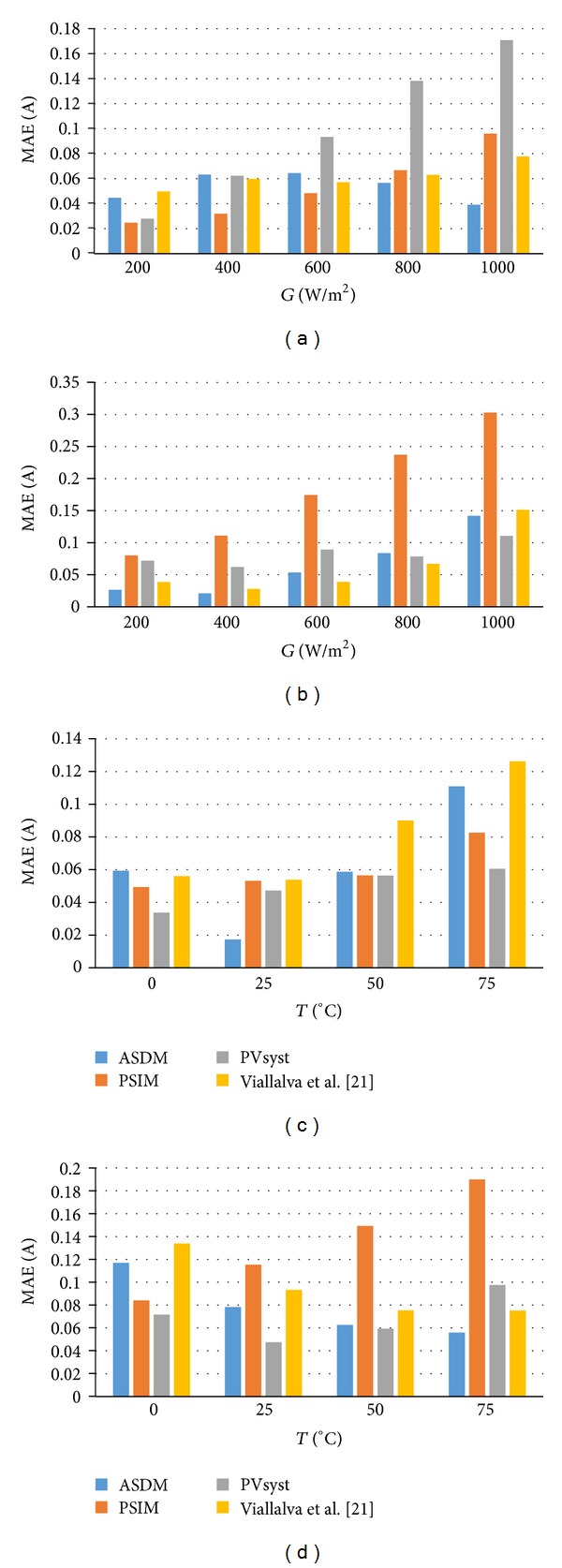
Mean absolute errors of the PV models at different atmospheric conditions: (a) SQ150-PC; (b) MSX60; (c) KC200GT; (d) HIT 180.

In order to further evaluate the estimation performance of the ASDM, more exhaustive tests have been conducted on the tested modules. Figures 6(a) and 6(b) show the MAEs of the simulated results subjected to irradiance variation, and all measurements are performed at a temperature of 25°C. On the other hand, Figures 6(c) and 6(d) demonstrate the MAEs of the ASDM model for MSX60 and HIT Power 180 modules working at the same irradiance of 1000 W/m^2^ but at different temperatures. In Figure 6, it is evident that the ASDM model outperforms the commercial tools (PSIM and PVsyst) in most cases and obtains better fitness quality than Villalva's model at high irradiance and temperature levels.

Similar trend is observed in Tables 2 and 3, which show the REs of the calculated MPP locus at different operating conditions. In practical, predicting the locus of MPP is of importance in the improvement of power efficiency. For this reason, statistical analysis is conducted. Except for the tests on SQ150-PC module under high irradiance test condition, most REs of the ASDM are similar or even lower than those of others.

**Figure 7:**
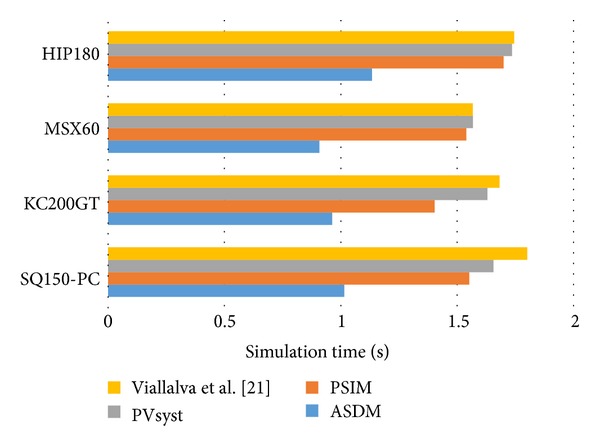
Simulation time of different PV models.

The simulation results described so far verify the accuracy of the proposed ASDM. Besides its low-error estimation performance, the ASDM has the advantage of deriving the *I-V* characteristics in closed form, and thus it supports high-speed computing.  Figure 7 makes a comparison among the efficiency of different PV models. In the tests, 10,000 operating points varied within the operating voltage range [0, *V*_oc_] are calculated in a general PC with a 2.40 GHZ Intel(R) Core (TM) 2 Duo CPU. It shows that the ASDM is able to reduce the simulation time by 30% compared to other tested models.

## 5. Conclusions

This paper has presented a simple approximate PV model which is capable of predicting the electrical characteristics of PV modules operating at a variety of atmospheric conditions. Continuous least squares approach is applied to fit the PV behaviors in a simple manner. The proposed mathematical modeling approach is easy and straightforward and doses not depend on iterative procedures to obtain solutions. The accuracy of the proposed model is evaluated through simulations. The results showed that the obtained current values are in good agreement with the experimental data. Future work integrating the real-time optimization of PV energy will highlight the value of this approximate modeling method.

## Figures and Tables

**Table 1:** Extracted ASDM parameters for different PV modules.

Module	*n*	*R*_*s*_ (Ω)	*R*_*p*_ (Ω)	*a*_0_	*a*_1_	RMSE
SQ150	1.6031	0.5334	808	0.9018	0.2877	2.10*E* − 03
KC200GT	1.1266	0.2764	206	0.6939	0.3771	5.29*E* − 02
MSX60	1.5390	0.1035	3140	0.9921	0.0843	6.23*E* − 04
HIT180	1.6240	0.4929	781	0.9753	0.1583	2.24*E* − 02

**Table 2:** Relative errors of the calculated *I*_mp_ at various irradiance levels.

Module	*G* (W/m^2^)	*T* (°C)	Relative error			
			ASDM	PSIM	PVsyst	Villalva et al. [21]
SQ150-PC	200	25	6.34%	0.51%	2.11%	1.51%
	400	25	3.29%	2.81%	0.30%	2.99%
	600	25	1.79%	3.60%	0.89%	2.68%
	800	25	1.18%	3.33%	0.29%	1.18%
	1000	25	0.59%	3.32%	0.00%	0.00%

KC200GT	200	25	1.93%	1.31%	0.39%	0.39%
	400	25	0.39%	2.90%	1.93%	1.93%
	600	25	0.00%	4.16%	1.15%	1.15%
	800	25	0.00%	5.48%	0.38%	0.38%
	1000	25	0.38%	6.81%	0.00%	0.00%

**Table 3:** Relative errors of the calculated *I*_mp_ at various temperature levels.

Module	*G* (W/m^2^)	*T* (°C)	Relative error			
			ASDM	PSIM	PVsyst	Villalva et al. [21]
MSX60	1000	0	1.59%	1.32%	1.59%	1.59%
	1000	25	0.00%	2.16%	0.00%	0.00%
	1000	50	1.31%	3.07%	1.31%	1.31%
	1000	75	3.70%	4.00%	3.70%	3.70%

HIT180	1000	0	2.11%	0.33%	0.35%	2.11%
	1000	25	0.00%	2.57%	2.59%	0.19%
	1000	50	0.82%	1.51%	0.41%	0.82%
	1000	75	0.22%	1.68%	1.12%	0.67%

## Appendix

### The Deduction Process of the Coefficients *a*_0_ and *a*_1_

According to (10), the linear normal equation for *e* ^*mI*^ can be rewritten in the following form:

(A.1) $$\begin{array}{c} a_{0}\int_{0}^{I_{\max}}dI+a_{1}\int_{0}^{I_{\max}}IdI=\int_{0}^{I_{\max}}e^{mI}dI,\\ a_{0}\int_{0}^{I_{\max}}IdI+a_{1}\int_{0}^{I_{\max}}I^{2}dI=\int_{0}^{I_{\max}}Ie^{mI}dI, \end{array}$$

where *I*_max⁡_ is the upper limit of the PV terminal current that is available at the *I-V* curves of the manufacturer's datasheet. After performing the integration, it yields

(A.2) $$\begin{array}{c} I_{\max}a_{0}+\frac{I_{\max}^{2}}{2}a_{1}=\frac{e^{mI_{\max}}}{m}-\frac{1}{m},\\ \frac{I_{\max}^{2}}{2}a_{0}+\frac{I_{\max}^{3}}{3}a_{1}=\frac{I_{\max}}{m}e^{mI_{\max}}-\frac{e^{mI_{\max}}}{m^{2}}+\frac{1}{m^{2}}. \end{array}$$

Equation (A.2) can be solved to obtain

(A.3) $$\begin{array}{c} a_{0}=-\frac{2}{mI_{\max}^{2}}\left[\left(I_{\max}-\frac{3}{m}\right)e^{mI_{\max}}+\left(2I_{\max}+\frac{3}{m}\right)\right],\\ a_{1}=\frac{12}{mI_{\max}^{3}}\left[\left(\frac{I_{\max}}{2}-\frac{1}{m}\right)e^{mI_{\max}}+\left(\frac{I_{\max}}{2}+\frac{1}{m}\right)\right]. \end{array}$$

Consequently, the least squares polynomial approximation of degree 1 for *I*_*d*_ is

(A.4) $$\begin{array}{c} I_{d}\left(I\right)≅I_{o}\left[e^{mV/R_{s}}\cdot \left(a_{0}+a_{1}I\right)-1\right]. \end{array}$$
